# A Reproducible Method for Isolation and *In Vitro* Culture of Functional Human Lymphoid Stromal Cells from Tonsils

**DOI:** 10.1371/journal.pone.0167555

**Published:** 2016-12-01

**Authors:** Yotam E. Bar-Ephraim, Tanja Konijn, Mehmet Gönültas, Reina E. Mebius, Rogier M. Reijmers

**Affiliations:** 1 Department of Molecular Cell Biology and Immunology, VU University Medical Center, Amsterdam, The Netherlands; 2 Department of Otolaryngology, MC Hospital, Amsterdam, The Netherlands; McGill University, CANADA

## Abstract

The stromal compartment of secondary lymphoid organs is classicaly known for providing a mechanical scaffold for the complex interactions between hematopoietic cells during immune activation as well as for providing a niche which is favorable for survival of lymphocytes. In recent years, it became increasingly clear that these cells also play an active role during such a response. Currently, knowledge of the interactions between human lymphoid stroma and hematopoietic cells is still lacking and most insight is based on murine systems. Although methods to isolate stromal cells from tonsils have been reported, data on stability in culture, characterization, and functional properties are lacking. Here, we describe a reproducible and easy method for isolation and *in vitro* culture of functional human lymphoid stromal cells from palatine tonsils. The cells isolated express markers and characteristics of T cell zone fibroblastic reticular cells (FRCs) and react to inflammatory stimuli by upregulating inflammatory cytokines and chemokines as well as adhesion molecules, as previously described for mouse lymphoid stroma. Also, cultured tonsil stromal cells support survival of human innate lymphoid cells, showing that these stromal cells can function as bone fide FRCs, providing a favorable microenvironment for hematopoietic cells.

## Introduction

Secondary lymphoid organs (SLO) are one of the hallmarks of the mammalian immune system. Dispersed throughout the body, these tissues with a defined microanatomy and spatial organization allow for the proper control and initiation of innate and adaptive immune responses and facilitate the interaction between adaptive lymphocytes and antigen presenting cells [1]. As such, the stromal cell compartment provides a scaffold for these immune cell interactions, while also forming a niche which is favorable for survival and, in case of an adaptive response, proliferation of hematopoietic cells. This is done by offering structural support in combination with secretion of homeostatic and, when activated, pro-inflammatory mediators [2–6]. Advances made in the past decade to analyze the structured anatomy of SLO and to study how the diverse array of cell types is distributed, emphasized the importance of specialized locations in SLO that specifically support the variety of cell types and functions [7]. With the recent discovery of a stromal cell niche for mouse and human innate lymphoid cells (ILC) it is now evident that SLO harbor organized stromal microenvironments that support adaptive lymphocyte survival and function [8].

ILCs form a family of cells of lymphoid origin that do not express rearranged antigen receptors and can be divided into three subsets (type 1, 2 and 3 ILCs; ILC1, 2 and 3 resp.) based on expression of transcription factors and production of cytokines [9]. While only a specific population of ILC3, described as lymphoid tissue inducer cells, play a crucial role during the early development of most SLO, all ILC subsets have been identified in mature SLO where they constitute and maintain a minor but important population of effector cells [10–15]. Like T cells, ILCs are dependent on IL-7 for their survival in homeostasis [6, 16, 17] and have been shown to interact closely with stromal cells in SLO [8, 17, 18].

Despite the rarity of ILCs in SLO many laboratories have developed ways to isolate pure ILC subsets and set up culture systems to grow and expand them for subsequent analysis [11, 12, 19–21]. While in murine settings, stromal cell lines have been used in the past to culture ILCs and ILC-like cell lines [22–24], human culture systems typically depend on recombinant cytokines (e.g. IL-7 and SCF or IL-2) in the presence or absence of hematopoietic feeder cell lines. To mimic the behavior of ILCs in human SLO more faithfully and to be able to study the role of ILCs in these organs, we set out to isolate and culture human SLO stroma *in vitro*. To this end we used cells isolated from resected palatine tonsils, which are readily available.

Here, we show that tonsil stromal cells (TSC) can be cultured as cell lines, while maintaining their functional phenotype for at least 8 passages and can be used in experiments as previously shown for murine SLO stroma [25]. Additionally we show that the TSC lines we cultured support survival of human ILCs. This will allow the study of human hematopoietic cells in an environment that more faithfully mimics the microenvironment they encounter *in vivo*.

## Materials and Methods

### Tonsils

Palatine tonsils were obtained from the department of Otolaryngology, MC Slotervaart, Amsterdam, the Netherlands. Anonymous collection of tonsil tissue was approved by the Medical Ethical Committee of the VU University Medical Center (Amsterdam, The Netherlands), in accordance with the Declaration of Helsinki and according to Dutch law. As tissue was collected anonymously, no signed informed consent was required.

### Flow cytometry and antibodies

For flow cytometric analysis, cells were detached with trypsin and kept in a single cell suspension in PBS supplemented with 2% (*v/v*) heat inactivated (h.i.) new born calf serum (NBCS) on ice to prevent re-attaching.

The following antibodies were used: rat-anti-human podoplanin (PDPN, clone NZ-1, AngioBio, San Diego, CA, USA), mouse-anti-human CD31-AlexaFluor^®^647 (clone WM59, BD eBiosciences, Mountain View, CA, USA), mouse-anti-human CD45-eFluor^®^ 450 (clone 2D1, eBioscience, San Diego, CA, USA). Exclusion of dead cells was done using staining with Fixable Viability Dye eFluor^®^ 780 (ebioscience, San Diego, CA, USA). Detection of unlabeled antibodies was done with goat-anti-rat AlexaFluor^®^ 488 or 555 (Thermo Fischer Scientific, Waltham, MA, USA).

Analysis was performed using a LSR-Fortessa X20 (BD Bioscience, Mountain View, CA, USA) flow cytometer. Further analysis was done using Flowjo Software version 10 for Microsoft (Tree Star, San Carlos, CA). Gating was done based on Fluorescence Minus One (FMO) controls.

### FACS sorting of ILCs and co-culture

TSC lines were trypsinized, replated in 96 well plates in a density of 20000–30000 cells per well and left to re-attach over night at 37°C.

Tonsil tissue was cut into small pieces and incubated for 30 min at 37°C in PBS supplemented with 0.5 mg/mL Collagenase D (Roche Life Sciences, Almere, the Netherlands). The tissue was subsequently disrupted mechanically and mononuclear cells were further isolated by filtering over a 700020μm nylon cell strainer (Falcon) and gradient centrifugation on lymphoprep (d = 1.077, Fresenius Kab, Berg i Østfold, Norway).

The mononuclear cell fraction was then enriched for CD117^+^ cells using the CD117 MicroBead kit (Milteryi Biotec, Bergisch Gladbach, Germany) according to manufacturer’s protocol. For ILC sorting the cells were stained with the following antibodies: mouse-anti-human CD3 (UCHT1), mouse-anti-human CD11c (3.9), mouse-anti-human CD19 (HIB19), mouse-anti-human CD14 (61D3), mouse-anti-human CD34 (4H11), mouse-anti-human CD94 (DX22, all FITC labeled), mouse-anti-human CD127-PE or APC (eBioRDR5, all Ebioscience, San Diego, CA, USA) and CD117- PE-eVio770 (A3C6E2, Miltenyi Biotec, Bergisch Gladbach, Germany). Cells were finally sorted using a MoFlo cell sorter (Beckman Coulter Inc., Brea, CA, USA). Sorted ILCs were co-cultured for 4 days with or without TSC, 50 ng/mL recombinant human (rh) IL-7 (Peprotech, Rocky Hill, NJ, USA) in DMEM supplemented with 10% (*v/v*) h.i. FCS, penicillin, streptomycin and L-glutamine.

### In vitro stimulation of TSC lines

TSC lines were trypsinized, re-plated in 6 well plates and cultured at 37°C. When confluent, cells were stimulated with either rhIFNγ or rhTNFα (both Peprotech, Rocky Hill, NJ, USA) for 6 hours.

### RNA isolation and real-time PCR

Cells were homogenized in Trizol reagent (Thermo Fischer Scientific, Waltham, MA, USA). RNA was isolated by centrifugation in Phase Lock Gel-heavy 1.5mL tubes (5 Prime GmbH, Hamburg, Germany), subsequently precipitated in 2-propanol (Sigma Aldrich, St. Louis, MO, USA) and finally washed with 75% EtOH (Cargill, Schiphol, the Netherlands). cDNA was synthesized from total RNA using RevertAid First Strand cDNA Synthesis Kit (Fermentas Life Sciences, Burlington, Canada) according to manufacturer's protocol.

mRNA isolation from small cell numbers upon co-culture experiments was done using mRNA capture kit (Roche Life Sciences, Almere, the Netherlands) according to manufacturer’s protocol and cDNA was subsequently synthesized using Reverse Transcription System (Promega, Madison, WI, USA) according to manufacturer’s protocol.

Real-time (rt)PCR was performed using SYBR Green mastermix (Foster City, CA, USA) on StepOne real-time PCR systems from Applied Biosystems (Bleiswijk, The Netherlands). Used primer sequences are listed in Table 1.

**Table 1:** Used rtPCR primer sequences.

Gene	Forward Primer	Reverse Primer
GAPDH	CCA TGT TCG TCA TGG GTG TG	GGT GCT AAG CAG TTG GTG GTG
IL-7	GCC TCC TTG GTG TCG TCC GC	AAC GCT TGG CGA CTG GGA GC
TRANCE	TCG AGG TCT CCA ACC CCT CCT	ACC GTT GGG GCC ATG CCT CT
LTBR	CCA GGC ACC TAT GTC TCA GCT A	TGG TCA GGT AGT TCC AGT GC
VCAM-1	TGA AGG ATG CGG GAG TAT ATG A	TTA AGG AGG ATG CAA AAT AGA GCA
ICAM-1	TAG CAG CCG CAG TCA TAA TGG G	AGG CGT GGC TTG TGT GTT CG
CCL21	GCA TGG CTG AGC TGC CCA CA	TGG GGT GTA CTG GGG AGC CG
CXCL10	GCATTCAAGGAGTACCTCTCTCT	TTGTAGCAATGATCTCAACACGT
CXCL12	CTC CAA ACT GTG CCC TTC AGA	CCT GAA TCC ACT TTA GCT TCG G
CXCL13	TCC CTA GAC GCT TCA TTG ATC GA	CAG CTT GAG GGT CCA CAC ACA CA
DCN	CCA GAA GTT CCT GAT GAC C	AGG TCT AGC AGA GTT GTG TC
BGN	GTC CTT TCG GCT GCC ACT G	GTA GAG GTG CTG GAG ACC C
COL1a1	CAC CGA CCA AGA AAC CAC C	CTG TCC AGG GAT GCC ATC TC
COL5a2	GAA GAC GAG GAT GAA GGA TAT GG	ACA CAG ATC TGA CAA GGG GC
FN1	CAA AGC AAG CCC GGT TGT TA	CCC ACT CGG TAA GTG TTC CC
FMOD	GTC AAC ACC AAC CTG GAG A	CTG CAG CTT GGA GAA GTT C
CEBPA	GTG CGT CTA AGA TGA GGG GG	CAT TGG AGC GGT GAG TTT GC
FAP	GAA CCA TGA AAA GTG TGA ATG CT	TGG ACG AGG AAG CTC ATT TCC
SPARC	CCA CTG AGG GTT CCC AGC	TAC CTC AGT CAC CTC TGC CA

### Immune fluorescence

For immunofluorescence analysis, tonsil tissue was embedded in OCT compound (Sakura Finetek, Leiden, the Netherlands), snap frozen in liquid nitrogen and kept at −80°C. Tissue sections (6 μm) were fixed with acetone for 10 min, air dried, and incubated in blocking buffer (PBS supplemented with 2% (*v/*v) h.i. NBCS and 5% (*v/*v) normal human serum). Samples were incubated with primary antibodies for 1–2 h in PBS containing 2% (*v/*v) h.i. NBCS and when needed, further incubated with appropriate secondary antibodies/reagents for 30 min. Sections were mounted in vynol with DAPI (Calbiochem). Images were acquired on Zeiss LSM 710 (Carl Zeiss) and further processed using Adobe Photo Shop and Illustrator CS6 software.

### Statistics

Results are given as the mean ± SEM or SD. Statistical analysis was done using GraphPad Prism 4 Software (La Jolla, CA, USA). Due to small sample size we could not assume normal distribution and/or equal variance and thus used either one- or two tailed Mann-Whitney tests for comparisons between two groups or Two Way ANOVA with Bonferonni’s correction for comparisons between multiple groups, as described in the figure legends. Significance is indicated by * p ≤ 0.05 or ** p < 0.01, *** p<0.001. We did not use statistical methods to predetermine sample size of human samples, nor were the investigators blinded to sample identity or results.

## Results

### Digestion and generation of tonsil stromal cell lines

Generation of tonsil stromal cell (TSC) lines was done according to methods previously reported for isolation of stromal cells from mouse and human lymph nodes [25]. In short, 8–10 pieces of approximately 5X5 mm were cut from different parts of tonsils in order to prevent selection bias (S1A and S1B Fig) and incubated in warm RPMI, supplemented with 0.6mg/mL collagenase P, 2.4mg/mL dispase and 0.3mg/mL DNAse I (S1C Fig). Every 5 min. tissue was re-suspended, supernatant was collected in PBS supplemented with 2% h.i. FCS and 5mM EDTA in a separate tube and fresh digestion medium was added to the tissue (S1D Fig). This process was repeated 4–5 times, after which the collected supernatant was centrifuged and the cells were suspended in RPMI-1640 supplemented with 10% (*v/v*) h.i. FCS, penicillin, streptomycin and L-glutamine and cultured o/n in collagen-coated culture flasks (S1E and S1F Fig). At day 1 and 2 post isolation the cells were extensively washed with PBS to wash away CD45^+^ cells after which the adherent cells were allowed to grow to confluency and passaged into new flasks (S1G Fig). For passaging, cells were detached from the flask by using 0.2% (*v/v*) trypsin supplemented with 5mM EDTA in PBS.

### Tonsils harbor characteristic lymphoid stromal cell subsets

**Fig 1:**
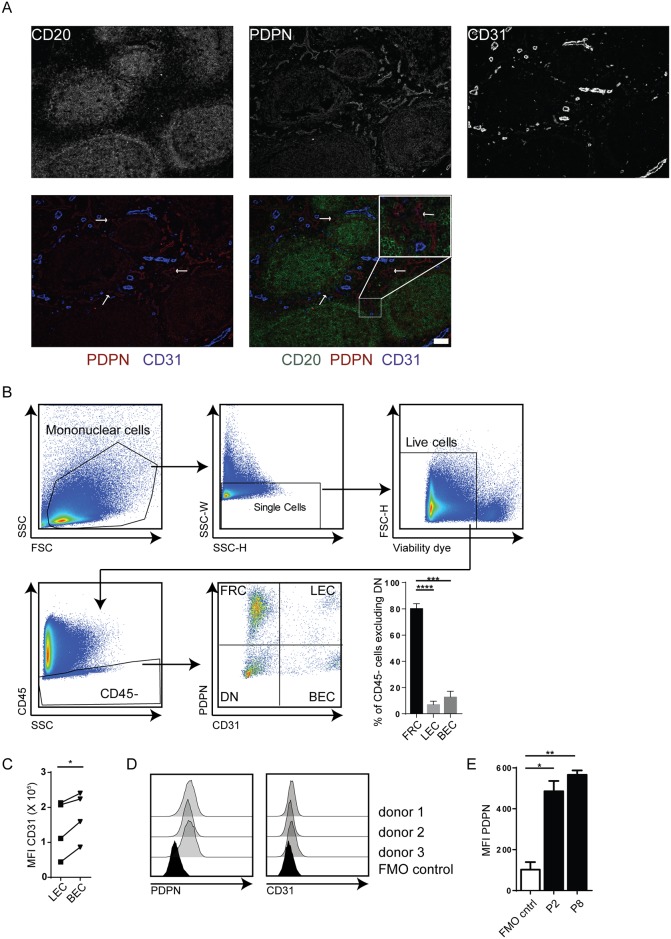
Isolated stromal cells show phenotypic characteristics of FRCs, LECs and BECs. (A) Representative immune fluorescence staining of tonsil tissue stained for CD20 (green), PDPN (red) and CD31 (blue). Bars represents 50μm for all images. (B) Gating strategy and *ex vivo* phenotype of isolated stomal cells. Mononuclear cells were gated based on FSC vs SSC profile, after which a gate was set on single cells. Dead cells were subsequently excluded using a viability dye staining and a gate was set for CD45^-^ cells. Finally, FRCs, LECs and BECs were defined by staining for PDPN and CD31. Plot shows percentages of FRCs (PDPN^+^CD31^-^), LECs (PDPN^+^CD31^+^) and BECs (PDPN^-^CD31^+^) within the CD45^-^ cells upon exclusion of DN stromal cells. Staining shown is representative for stainings of 5 different donors. ***: p < 0.001, ****: p < 0.0001, Two Way ANOVA with Turkey’s multiple comparison test. (C) Expression levels of CD31 on LECs in comparison to BECs. *: p<0.05, Wilcoxon matched-pairs signed rank test. (D) Phenotype of cultured cell lines. PDPN and CD31 staining of cells cultured for 2 passages of 3 independent donors. (E) PDPN expression through culturing. Shown are the MFI values of PDPN staining upon culture for 2 (P2) or 8 (P8) passages of 3 independent donors. *: p < 0.05, **: p < 0.001, Two Way ANOVA with Turkey’s multiple comparison test. For all plots: error bars represent mean ±SD.

Similar as in murine lymphoid tissue, CD45^-^ stromal subsets within the human tonsil can be distinguished by determining the expression of the protein markers PDPN and CD31 [26–28]. First, the presence of the cellular subsets was confirmed by staining frozen sections for podoplanin (PDPN) and CD31 (Fig 1A). For convenient orientation within the tissue, a B cell staining was included (CD20, Fig 1A). Dense fibroblastic reticular cell (FRC) staining (PDPN^+^) could be seen throughout the tonsil, which was even more pronounced within the B cell follicles. The interfollicular space was found to encompass blood and lymphatic vessels composed of CD31^+^ blood endothelial cells (BECs) and PDPN^+^CD31^+^ lymphatic endothelial cells (LECs) respectively (Fig 1A). Flow cytometric analysis of cells directly after enzymatic digestion revealed that the recovered stromal compartment was mainly formed by PDPN^+^CD31^-^ FRCs (80.22% ±3.72), while a smaller portion of the cells were either PDPN^+^CD31^+^ LECs (7% ±2.63) or PDPN^-^CD31^+^ BECs (12.78% ±4.43) (Fig 1B). Both immunofluorescence (Fig 1A) and flow cytometry (Fig 1B and 1C) revealed that human BECs expressed higher levels of CD31 when compared to LECs (Fig 1C).

Subsequently, tonsil stromal cell (TSC) lines were grown out by repeated passage. In contrast to a previous report considering culture of human LN stromal cells [25], these cultures were characterized and already after their second passage the cells that remained in culture were near uniformly CD45^-^PDPN^+^CD31^-^, corresponding to FRCs (Fig 1D). PDPN expression remained stable through culture for at least 8 passages (Fig 1E). These results show that the classically described stromal subsets for mouse and human lymph nodes can also be identified in human tonsils upon enzymatic digestion. The differential expression of PDPN and CD31 will allow for FACS-sorting of the stromal subsets FRCs, BECs, and LECs. However, upon bulk *ex vivo* culture (without FACS sorting), FRC-like cells dominate the cultures, which can be further cultured as cell lines for at least 8 passages.

### Isolated TSC lines resemble bone fide FRCs

**Fig 2:**
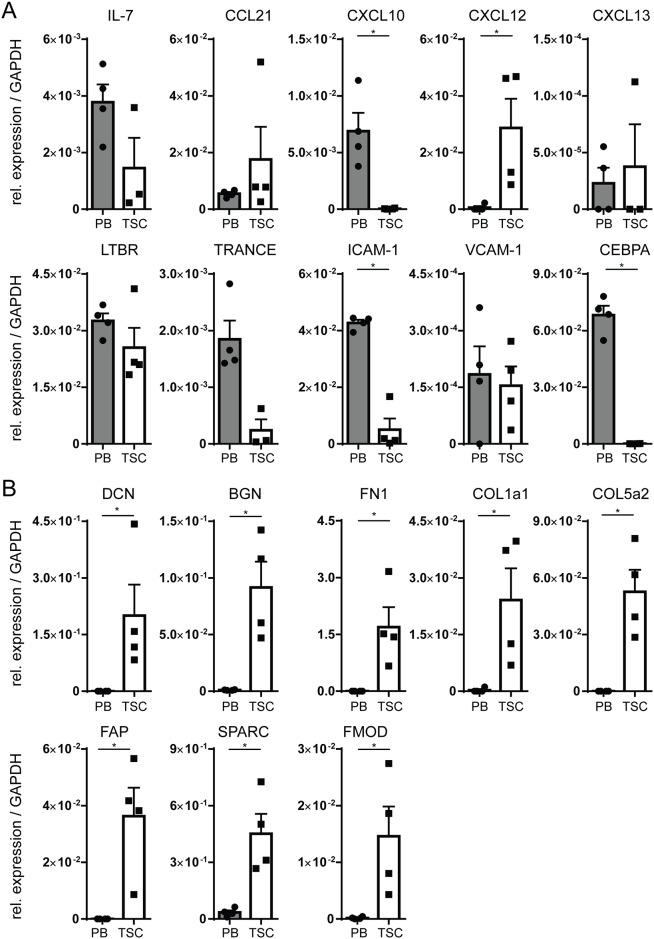
Transcript expression of stromal-associated genes by cultured TSC. (A) PCR analysis of cultured TSC (harvested at passage 2 or 3, n = 4 individual donors) as compared to PBMCs (n = 4 individual donors) for cytokines/ chemokines and their receptor, associated with lymphoid stromal cells. Shown is expression relative to GAPDH. (B) PCR analysis of cultured TSC (harvested at passage 2 or 3, n = 4 individual donors) as compared to PBMCs (n = 4 individual donors) for genes associated with extracellular matrix. Shown is expression relative to GAPDH. For all plots: *: p < 0.05, Two Tailed Mann-Whitney Test, error plots represent mean ± SEM.

To further characterize the TSC lines we performed PCR analysis for IL-7, CCL21, CXCL10, CXCL12, CXCL13, TRANCE, LTβR, ICAM-1, and VCAM-1, all of which are known to be produced by lymphoid stromal cells [29], and CEBPA, which is associated with myeloid cells [30] as a negative control. We used CD45^+^ PBMCs from healthy donors as controls. As expected and in accordance with previous reports, ICAM-1, IL-7, CXCL10, TRANCE and LTBR were found to be expressed in both stroma and PBMCs [31–34], while CXCL12 was found to be expressed by TSC and not by PBMCs [35] (Fig 2A). In line with its role in cells of the myeloid lineage [30], CEBPA was found to be expressed only in PBMCs, but not in stromal cells (Fig 2A).

Beside chemokines and cytokines, FRCs produce extracellular matrix (ECM) proteins that, for a large part, make up a reticular network known as the conduit system [29]. Accordingly, the cells cultured in our system also produced transcripts involved in the assembly of the conduit network, inculding decorin (DCN), biglycan (BGN), collagen type I (COL1A1), collagen type V (COL5A2), fibronectin 1 (FN1), focal adhesion protein (FAP), SPARC, and fibromodulin (FMOD) which were not expressed in the PBMC controls (Fig 2B). In summary, the stromal cell lines generated from tonsils showed phenotypic characteristics of genuine FRCs both at protein (Fig 1) and transcript (Fig 2) level [29].

### Isolated TSC lines remain stable in culture

**Fig 3:**
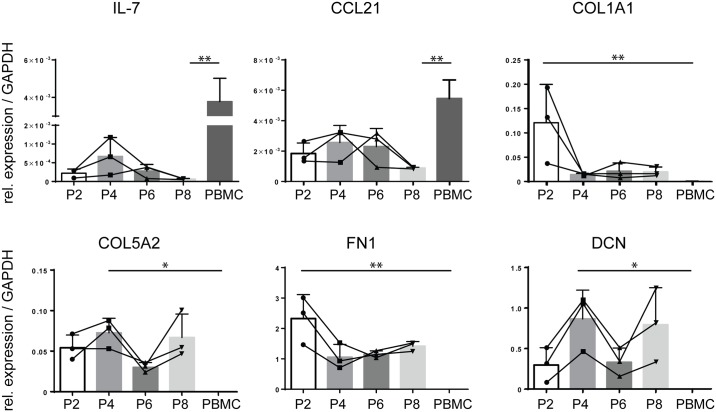
Transcript expression of stromal-associated genes by cultured TSC remains stable in culture. PCR analysis of cultured TSC harvested at passage 2, 4, 6 and 8 (P2,P4, P6 and P8, respectively) and PBMCs for IL-7, CCL21, COL1A1, COL5A2, FN1 and DCN (n = 3 individual donors for the stromal cells, n = 4 for PBMCs). *: p < 0.05, **: p < 0.01, Kruskall-Wallis test with Dunn’s multiple comparisons test. Error plots represent mean ± SD.

To determine whether the generated TSC lines remain phenotypically stable in culture, we analyzed gene expression for FRC signature genes in time. Indeed, like protein expression of PDPN (Fig 1E), expression of IL-7, CCL21, COL1A1, COL5A2, FN1 and DCN transcripts did not differ between cells harvested at P2, P4, P6 or P8 (Fig 3). Although some fluctuation was seen between different passages, in all passages FRC specific genes COL1A1, COL5A2, FN1 and DCN were higher than in PB. These results show that the FRC phenotype remains stable in culture for at least 8 passages.

### TSC lines up-regulate CXCL10, ICAM-1 and VCAM-1 upon inflammatory cytokine stimulation

**Fig 4:**
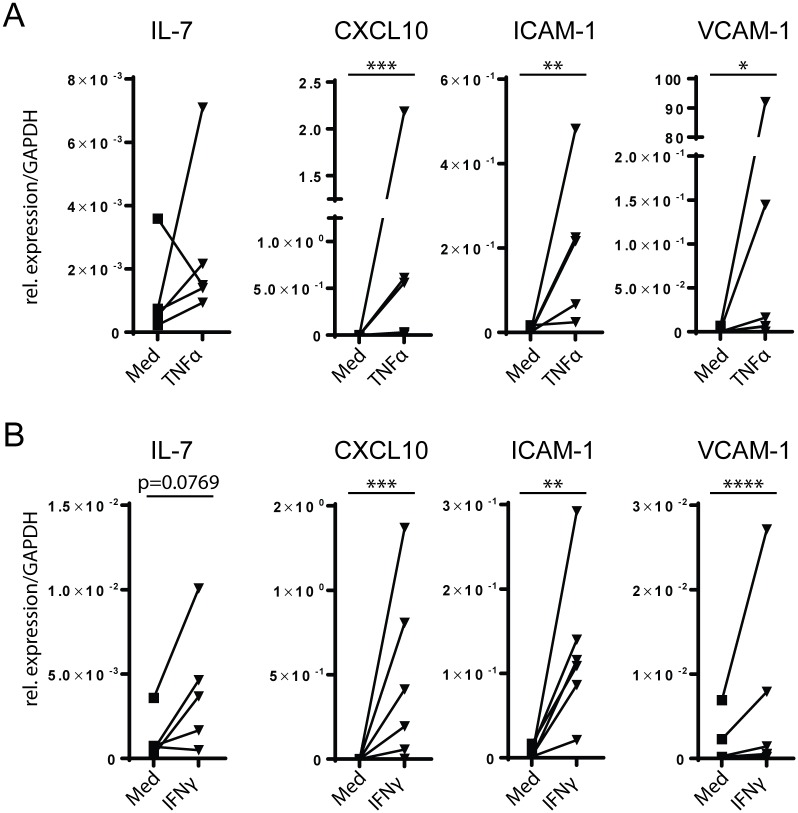
Stimulation of TSC lines with pro-inflammatory cytokines. (A) TSC (passage 2 or 3, n = 6 individual donors) were cultured for 6 hr with either 5ng/mL rhTNFα or medium alone. Shown is expression relative to GAPDH. (B) TSC (passage 2 or 3, n = 6 individual donors) were cultured for 6 hr with either 300U/mL rhIFNγ or medium alone. Shown is expression relative to GAPDH. For all plots: *: p < 0.05, **: p < 0.01, ***: p < 0.001, ****: p < 0.0001, ratio-paired Student’s T-test.

In case of immune activation, stromal cells in mouse SLO take an active part in forming the proper microenvironment for the ongoing immune response, including up-regulation of adhesion molecules and secretion of inflammatory cytokines [29, 36]. To assess whether human TSC do the same, we stimulated TSC lines with either 5ng/mL rhTNFα or 300U/mL rhIFNγ for 6 hours and assessed expression of stromal cell-associated genes by quantitative RT-PCR. Both IFNγ and TNFα led to an up-regulation in all donors of CXCL10, ICAM-1 and VCAM-1, while an upregulation of IL-7 in all but one donor was only seen upon stimulation with IFNγ (Fig 4). In contrast, expression levels of LTBR, TRANCE, CXCL12, CXCL13 and the ECM-associated genes were unaffected by either stimuli (data not shown).

These data indicate that, like primary mouse and human stromal cells, our TSC lines regulate transcripts for adhesion molecules, pro-inflammatory cytokines, and chemokines upon encounter with inflammatory stimuli, which may facilitate an ongoing immune response *in vivo*, as these molecules can control hematopoietic cell adhesion, migration, and function.

### TSC lines support survival of innate lymphoid cells

**Fig 5:**
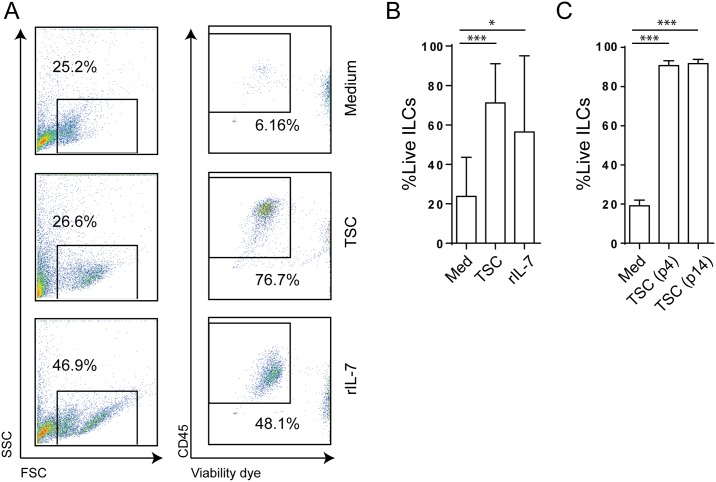
Cultured TSC lines support ILC survival *in vitro*. (A) FACS sorted ILCs were co-cultured with either TSC (harvested at passage 2 or 3), 50ng/mL rhIL-7 or medium alone for 4 days. Shown are representative plots of FSC-A vs SSC-A profile as well as staining for CD45 and fixable viability marker to assess ILC viability upon culture. Plots are representative for 4 independent experiments. (B) Percentages of viable ILCs upon co-culture with either TSC line, rhIL-7 or medium alone for 4 days. n = 5 individual donors. (C) FACS sorted ILCs were co-cultured with TSC harvested at passage 4, TSC harvested at passage 14 or without stroma for 4 days. Shown are percentages of viable ILCs at day 4. n = 3 individual donors. For all plots: *: p<0.05, ***: p<0.001, Two way ANOVA with Bonferroni correction. Error bars represent mean ± SD.

In steady state, SLO stroma creates a niche favorable for survival of lymphocytes including which innate lymphoid cells (ILCs). ILCs have been shown to reside in both human and mouse SLO mainly in the interfollicular region, where they associate with PDPN^+^CD31^-^TRANCE^+^ stromal cells [8, 18]. To address whether the TSC lines we grew out could support ILC survival, we FACS-sorted human ILCs from both peripheral blood and tonsils (sort strategy and reanalysis in S2 Fig) and cultured them for 4 days with or without TSC and compared these cultures to sorted ILCs cultured with rhIL-7 as a positive control. As expected, ILCs cultured with TSC showed increased survival as compared to cells cultured without stroma, similar to ILCs cultured in rhIL-7 alone (Fig 5A and 5B). Finally, to establish functional stability of the TSC, we cultured ILCs for four days with TSC from passage 4 or 14 from the same donor. In both early (passage 4) and late (passage 14) passages, TSC supported ILC survival as opposed to no stroma control (Fig 5C).

Collectively, we describe a method to isolate human tonsil-derived stromal cells and to grow out stable cell lines that support ILC survival *in vitro*. This provides means to study human ILCs in a microenvironment which more faithfully resembles their natural milieu in SLO.

## Discussion

In this report, we show for the first time that stromal cells isolated and grown out of palatine tonsils can be stably cultured *in vitro* as cell lines and function as *bone fide* FRCs. Biomedical research in human settings is inherently restricted due to the limitation to *ex vivo* or *in vitro* models. It is also widely accepted that *in vivo* experiments carried out in animal models are not always completely representative for human biology. As a consequence, there is a great need for model systems which mimic the human *in vivo* situation as accurately as possible. Currently, assays modeling the human immune response are often done *in vitro*, using only hematopoietic cells. Doing so, the role of the stromal compartment of the lymphoid tissue in such a response is ignored. At the same time, it has become exceedingly clear in the past two decades that the SLO stroma is taking active part in the regulation of the immune responses rather than only providing a structural scaffold for interactions between hematopoietic cells [3, 37, 38]. For example, production of CCL19 and 21 enhances motility [39–42] and survival [6] of T cells as well as maturation and antigen uptake by DCs [43–45], while IL-7 has been shown to enhance TCR signaling, also augmenting effector function and T cell memory formation [6, 46]. Conversely, via production of iNOS [47] and presentation of peripheral tissue antigens (PTA) in the context of MHC-II [48–54], SLO stroma has been shown to attenuate T cell responses by direct suppression or maintenance of Tregs. Both the stimulating and inhibiting roles of the stroma in the context of an immune response can greatly influence the outcome of such a response and should be taken into account when investigating it. With our isolation and culture method, human tonsil stroma can be easily and reproducibly isolated and cultured for use in different experimental settings.

The TSC lines described here express homeostatic chemokines and cytokines as well as components of the ECM, as described for mouse SLO stroma [25, 29]. Additionally, the cells support survival of ILCs *in vitro* which is in line with the role of stroma in creating a favorable niche for lymphocyte survival within SLOs [3]. On the flip side, human ILCs have been shown in the past to cause induction of ICAM-1 and VCAM-1 on stromal cells, as a consequence of LTBR and/or TNFR signaling [55]. This ILC-stromal cell interaction, which is long known from lymphorganogenesis during ontogeny [56] and regeneration of lymphoid tissue following LCMV infection in mice [57] may also play a role in the formation of lymphoid aggregates in cancer or autoimmune diseases [55, 58, 59]. Although many other cell types including adaptive lymphocytes express lymphotoxin and TNFα, the permanent presence of ILCs in human SLOs makes it tempting to speculate that these cells play a role in the maintenance of the homeostasis of SLO stroma.

Maintaining survival of lymphocytes is also important in the context of lymphomas. Human stromal cells have been shown to support survival of malignant B cells *in vitro* [26] and different stromal gene signatures, including expression levels of various ECM transcripts, have been shown to correlate with different survival rates of diffuse large B cell lymphoma patients [60]. A favorable niche for survival of malignant cells which is created by survival factors secreted by SLO stroma can thus greatly influence the efficacy of treatment regiments of patients. Standardization of culture systems for functional human stromal cells as shown here can boost research into these stromal cell-malignant lymphocyte interactions and the influence these have on patient treatment.

Of note, the cells we use here are derived from tonsils rather than from lymph nodes, which have been shown to grow for a short period of time *in vitro* as well [25]. Also, we provide here a functional characterization of these cells, showing they remain stable in culture for at least 8 passages, which readily allows for *in vitro* gene manipulation by *eg*. Cas9/CRISPR techniques. In addition, tonsils are different from lymph nodes in a number of important aspects. Most importantly, resected tonsils are often inflamed, which can alter the phenotype of the isolated cells [28, 29]. However, the cells did react to stimulation with either IFNγ or TNFα, implying culturing allowed the cells to return to a homeostatic state (Fig 3). Also, tonsils lack afferent lymphatics and have lymphatic sinuses that are positioned directly below the squamous epithelium [61], which can have an effect on the stromal populations isolated and makes it more challenging to isolate and culture LECs. Indeed, in contrast to what has been shown for stromal cells isolated from human LNs, already at early time points (passage 1–2) we did not observe any endothelial cells in our culture. This suggests that the FRC-like cells out-grow the endothelial cells when cultured together. Based on our *ex vivo* flow cytometry data, however, it is likely that provided cell sorting performed directly upon tonsil digestion, the isolation method described here will allow for the purification of all typical stromal cell types (including LECs and BECs). Finally, an advantage of using tonsils as source for stromal cells is the availability of tissue. As tonsillectomies are routinely performed in many hospitals, tonsils are much more readily available than human lymph nodes which are often isolated post-mortem.

In conclusion, we show here that isolated stromal cells from tonsils can grow in culture as stable and *bone fide* FRCs cell lines. Our culture system offers a reliable, reproducible method for deeper investigations of interactions between human lymphoid tissue stroma and healthy or malignant hematopoietic cells, and to more closely and faithfully recapitulate the complex and multifunctional *in vivo* microenvironment of SLOs.

## Supporting Information

S1 Fig Method of isolation of tonsil derived stromal cells. (A) 8–10 pieces of about 0.5X0.5 cm were cut from different parts of tonsils (B). The pieces of tonsil were incubated in RPMI supplemented with 0.6mg/mL collagenase P, 2.4mg/mL dispase and 0.3mg/mL DNAse I for 5 min. (C) after which they were disrupted using a 1000μL pipette (D). Subsequently, the supernatant was transferred to a tube containing PBS supplemented with 1% hi FCS and 2mM EDTA and centrifuged (E). Fresh enzyme containing medium was added to the pieces of tonsils and the process was repeated 4–5 times. Finally, the cells were centrifuged, taken up in culture medium and cultured in a collagen-coated T75 culture flask at 37°C / 5%CO_2_ (F). (G) Images of cultured tonsil cell suspensions 24hr. upon isolation before (left) and after (right) extensive washing with PBS. Images are representative of 13 independent isolations.
(TIF)

S2 Fig FACS sorting of human innate lymphoid cells. (A) ILCs were sort purified from cKit+ enriched fractions of human tonsils or peripheral blood as follows: lymphocytes were gated based on FSC-A vs SSC-A profile, after which doublets were excluded. Lin^-^ cells were subsequently gated and finaly ILC3 were sorted as CD127^+^cKit^+^ cells. The lineage cocktail included monoclonal antibodies for human CD3, CD11c, CD14, CD19, CD34 and CD94. (B) Reanalysis of sorted cells. Images are representative of 5 independent sorts. In all plots: numbers represent percentage of cells within gate as part of all cells in the plot.
(TIF)

## References

[1] Katakai T, Hara T, Lee JH, Gonda H, Sugai M, Shimizu A. A novel reticular stromal structure in lymph node cortex: an immuno-platform for interactions among dendritic cells, T cells and B cells. Int Immunol. 2004;16(8):1133–42. 10.1093/intimm/dxh113 15237106

[2] Bajenoff M, Egen JG, Koo LY, Laugier JP, Brau F, Glaichenhaus N, et al Stromal cell networks regulate lymphocyte entry, migration, and territoriality in lymph nodes. Immunity. 2006;25(6):989–1001. 10.1016/j.immuni.2006.10.011 17112751 PMC2692293

[3] Mueller SN, Germain RN. Stromal cell contributions to the homeostasis and functionality of the immune system. Nat Rev Immunol. 2009;9(9):618–29. 10.1038/nri2588 19644499 PMC2785037

[4] Roozendaal R, Mebius RE. Stromal cell-immune cell interactions. Annu Rev Immunol. 2011;29:23–43. 10.1146/annurev-immunol-031210-101357 21073333

[5] Koning JJ, Mebius RE. Interdependence of stromal and immune cells for lymph node function. Trends Immunol. 2012;33(6):264–70. 10.1016/j.it.2011.10.006 22153930

[6] Link A, Vogt TK, Favre S, Britschgi MR, Acha-Orbea H, Hinz B, et al Fibroblastic reticular cells in lymph nodes regulate the homeostasis of naive T cells. Nat Immunol. 2007;8(11):1255–65. 10.1038/ni1513 17893676

[7] Qi H, Kastenmuller W, Germain RN. Spatiotemporal basis of innate and adaptive immunity in secondary lymphoid tissue. Annu Rev Cell Dev Biol. 2014;30:141–67. 10.1146/annurev-cellbio-100913-013254 25150013

[8] Hoorweg K, Narang P, Li Z, Thuery A, Papazian N, Withers DR, et al A Stromal Cell Niche for Human and Mouse Type 3 Innate Lymphoid Cells. J Immunol. 2015;195(9):4257–63. 10.4049/jimmunol.1402584 26378073 PMC4640183

[9] Artis D, Spits H. The biology of innate lymphoid cells. Nature. 2015;517(7534):293–301. 10.1038/nature14189 25592534

[10] Mackley EC, Houston S, Marriott CL, Halford EE, Lucas B, Cerovic V, et al CCR7-dependent trafficking of RORgamma(+) ILCs creates a unique microenvironment within mucosal draining lymph nodes. Nat Commun. 2015;6:5862 10.1038/ncomms6862 25575242 PMC4354100

[11] Cupedo T, Crellin NK, Papazian N, Rombouts EJ, Weijer K, Grogan JL, et al Human fetal lymphoid tissue-inducer cells are interleukin 17-producing precursors to RORC+ CD127+ natural killer-like cells. Nat Immunol. 2009;10(1):66–74. 10.1038/ni.1668 19029905

[12] Mjosberg JM, Trifari S, Crellin NK, Peters CP, van Drunen CM, Piet B, et al Human IL-25- and IL-33-responsive type 2 innate lymphoid cells are defined by expression of CRTH2 and CD161. Nat Immunol. 2011;12(11):1055–62. 10.1038/ni.2104 21909091

[13] Bernink JH, Peters CP, Munneke M, te Velde AA, Meijer SL, Weijer K, et al Human type 1 innate lymphoid cells accumulate in inflamed mucosal tissues. Nat Immunol. 2013;14(3):221–9. 10.1038/ni.2534 23334791

[14] Bar-Ephraim YE, Mebius RE. Innate lymphoid cells in secondary lymphoid organs. Immunol Rev. 2016;271(1):185–99. 10.1111/imr.12407 27088915

[15] Cornelissen F, Aparicio Domingo P, Reijmers RM, Cupedo T. Activation and effector functions of human RORC+ innate lymphoid cells. Curr Opin Immunol. 2011;23(3):361–7. 10.1016/j.coi.2011.03.002 21561752

[16] Schmutz S, Bosco N, Chappaz S, Boyman O, Acha-Orbea H, Ceredig R, et al Cutting edge: IL-7 regulates the peripheral pool of adult ROR gamma+ lymphoid tissue inducer cells. J Immunol. 2009;183(4):2217–21. 10.4049/jimmunol.0802911 19635901

[17] Hou TZ, Mustafa MZ, Flavell SJ, Barrington F, Jenkinson EJ, Anderson G, et al Splenic stromal cells mediate IL-7 independent adult lymphoid tissue inducer cell survival. Eur J Immunol. 2010;40(2):359–65. 10.1002/eji.200939776 19950181

[18] Kim MY, McConnell FM, Gaspal FM, White A, Glanville SH, Bekiaris V, et al Function of CD4+CD3- cells in relation to B- and T-zone stroma in spleen. Blood. 2007;109(4):1602–10. 10.1182/blood-2006-04-018465 17018858

[19] Crellin NK, Trifari S, Kaplan CD, Satoh-Takayama N, Di Santo JP, Spits H. Regulation of cytokine secretion in human CD127(+) LTi-like innate lymphoid cells by Toll-like receptor 2. Immunity. 2010;33(5):752–64. 10.1016/j.immuni.2010.10.012 21055975

[20] Allan DS, Kirkham CL, Aguilar OA, Qu LC, Chen P, Fine JH, et al An in vitro model of innate lymphoid cell function and differentiation. Mucosal Immunol. 2015;8(2):340–51. 10.1038/mi.2014.71 25138665

[21] Hoorweg K, Peters CP, Cornelissen F, Aparicio-Domingo P, Papazian N, Kazemier G, et al Functional Differences between Human NKp44(-) and NKp44(+) RORC(+) Innate Lymphoid Cells. Front Immunol. 2012;3:72 10.3389/fimmu.2012.00072 22566953 PMC3342004

[22] Possot C, Schmutz S, Chea S, Boucontet L, Louise A, Cumano A, et al Notch signaling is necessary for adult, but not fetal, development of RORgammat(+) innate lymphoid cells. Nat Immunol. 2011;12(10):949–58. 10.1038/ni.2105 21909092

[23] Tang Q, Ahn YO, Southern P, Blazar BR, Miller JS, Verneris MR. Development of IL-22-producing NK lineage cells from umbilical cord blood hematopoietic stem cells in the absence of secondary lymphoid tissue. Blood. 2011;117(15):4052–5. 10.1182/blood-2010-09-303081 21310921 PMC3087531

[24] Grzywacz B, Kataria N, Sikora M, Oostendorp RA, Dzierzak EA, Blazar BR, et al Coordinated acquisition of inhibitory and activating receptors and functional properties by developing human natural killer cells. Blood. 2006;108(12):3824–33. 10.1182/blood-2006-04-020198 16902150 PMC1895469

[25] Fletcher AL, Malhotra D, Acton SE, Lukacs-Kornek V, Bellemare-Pelletier A, Curry M, et al Reproducible isolation of lymph node stromal cells reveals site-dependent differences in fibroblastic reticular cells. Front Immunol. 2011;2:35 10.3389/fimmu.2011.00035 22566825 PMC3342056

[26] Ame-Thomas P, Maby-El Hajjami H, Monvoisin C, Jean R, Monnier D, Caulet-Maugendre S, et al Human mesenchymal stem cells isolated from bone marrow and lymphoid organs support tumor B-cell growth: role of stromal cells in follicular lymphoma pathogenesis. Blood. 2007;109(2):693–702. 10.1182/blood-2006-05-020800 16985173

[27] Garrafa E, Alessandri G, Benetti A, Turetta D, Corradi A, Cantoni AM, et al Isolation and characterization of lymphatic microvascular endothelial cells from human tonsils. J Cell Physiol. 2006;207(1):107–13. 10.1002/jcp.20537 16261591

[28] Mogoanta CA, Ion DA, Budu V, Mutiu G, Salplahta D, Afrem E. Evaluation of microvascular density in inflammatory lesions and carcinoma of palatine tonsil. Rom J Morphol Embryol. 2013;54(1):179–85. 23529327

[29] Malhotra D, Fletcher AL, Astarita J, Lukacs-Kornek V, Tayalia P, Gonzalez SF, et al Transcriptional profiling of stroma from inflamed and resting lymph nodes defines immunological hallmarks. Nat Immunol. 2012;13(5):499–510. 10.1038/ni.2262 22466668 PMC3366863

[30] Friedman AD. C/EBPalpha in normal and malignant myelopoiesis. Int J Hematol. 2015;101(4):330–41. 10.1007/s12185-015-1764-6 25753223 PMC4696001

[31] Lee TV, Kim DK, Peoples GE, Castilleja A, Murray JL, Gershenson DM, et al Secretion of CXC chemokine IP-10 by peripheral blood mononuclear cells from healthy donors and breast cancer patients stimulated with HER-2 peptides. J Interferon Cytokine Res. 2000;20(4):391–401. 10.1089/107999000312333 10805374

[32] Romme Christensen J, Bornsen L, Hesse D, Krakauer M, Sorensen PS, Sondergaard HB, et al Cellular sources of dysregulated cytokines in relapsing-remitting multiple sclerosis. J Neuroinflammation. 2012;9:215 10.1186/1742-2094-9-215 22978757 PMC3503813

[33] Most J, Schwaeble W, Drach J, Sommerauer A, Dierich MP. Regulation of the expression of ICAM-1 on human monocytes and monocytic tumor cell lines. J Immunol. 1992;148(6):1635–42. 1347304

[34] Locksley RM, Killeen N, Lenardo MJ. The TNF and TNF receptor superfamilies: integrating mammalian biology. Cell. 2001;104(4):487–501. 11239407 10.1016/s0092-8674(01)00237-9

[35] Nagasawa T, Tachibana K, Kishimoto T. A novel CXC chemokine PBSF/SDF-1 and its receptor CXCR4: their functions in development, hematopoiesis and HIV infection. Semin Immunol. 1998;10(3):179–85. 10.1006/smim.1998.0128 9653044

[36] Malhotra D, Fletcher AL, Turley SJ. Stromal and hematopoietic cells in secondary lymphoid organs: partners in immunity. Immunol Rev. 2013;251(1):160–76. 10.1111/imr.12023 23278748 PMC3539229

[37] Siegert S, Luther SA. Positive and negative regulation of T cell responses by fibroblastic reticular cells within paracortical regions of lymph nodes. Front Immunol. 2012;3:285 10.3389/fimmu.2012.00285 22973278 PMC3438460

[38] Chang JE, Turley SJ. Stromal infrastructure of the lymph node and coordination of immunity. Trends Immunol. 2015;36(1):30–9. 10.1016/j.it.2014.11.003 25499856

[39] Stachowiak AN, Wang Y, Huang YC, Irvine DJ. Homeostatic lymphoid chemokines synergize with adhesion ligands to trigger T and B lymphocyte chemokinesis. J Immunol. 2006;177(4):2340–8. 16887995 10.4049/jimmunol.177.4.2340

[40] Okada T, Cyster JG. CC chemokine receptor 7 contributes to Gi-dependent T cell motility in the lymph node. J Immunol. 2007;178(5):2973–8. 17312142 10.4049/jimmunol.178.5.2973

[41] Asperti-Boursin F, Real E, Bismuth G, Trautmann A, Donnadieu E. CCR7 ligands control basal T cell motility within lymph node slices in a phosphoinositide 3-kinase-independent manner. J Exp Med. 2007;204(5):1167–79. 10.1084/jem.20062079 17485513 PMC2118589

[42] Worbs T, Mempel TR, Bolter J, von Andrian UH, Forster R. CCR7 ligands stimulate the intranodal motility of T lymphocytes in vivo. J Exp Med. 2007;204(3):489–95. 10.1084/jem.20061706 17325198 PMC2137901

[43] Marsland BJ, Battig P, Bauer M, Ruedl C, Lassing U, Beerli RR, et al CCL19 and CCL21 induce a potent proinflammatory differentiation program in licensed dendritic cells. Immunity. 2005;22(4):493–505. 10.1016/j.immuni.2005.02.010 15845453

[44] Yanagawa Y, Onoe K. CCL19 induces rapid dendritic extension of murine dendritic cells. Blood. 2002;100(6):1948–56. 10.1182/blood-2002-01-0260 12200351

[45] Yanagawa Y, Onoe K. CCR7 ligands induce rapid endocytosis in mature dendritic cells with concomitant up-regulation of Cdc42 and Rac activities. Blood. 2003;101(12):4923–9. 10.1182/blood-2002-11-3474 12609829

[46] Pellegrini M, Calzascia T, Toe JG, Preston SP, Lin AE, Elford AR, et al IL-7 engages multiple mechanisms to overcome chronic viral infection and limit organ pathology. Cell. 2011;144(4):601–13. 10.1016/j.cell.2011.01.011 21295337

[47] Siegert S, Huang HY, Yang CY, Scarpellino L, Carrie L, Essex S, et al Fibroblastic reticular cells from lymph nodes attenuate T cell expansion by producing nitric oxide. PLoS One. 2011;6(11):e27618 10.1371/journal.pone.0027618 22110693 PMC3215737

[48] Fletcher AL, Lukacs-Kornek V, Reynoso ED, Pinner SE, Bellemare-Pelletier A, Curry MS, et al Lymph node fibroblastic reticular cells directly present peripheral tissue antigen under steady-state and inflammatory conditions. J Exp Med. 2010;207(4):689–97. 10.1084/jem.20092642 20308362 PMC2856033

[49] Baptista AP, Roozendaal R, Reijmers RM, Koning JJ, Unger WW, Greuter M, et al Lymph node stromal cells constrain immunity via MHC class II self-antigen presentation. Elife. 2014;3. 10.7554/eLife.04433 PMC4270074 25407678

[50] Magnusson FC, Liblau RS, von Boehmer H, Pittet MJ, Lee JW, Turley SJ, et al Direct presentation of antigen by lymph node stromal cells protects against CD8 T-cell-mediated intestinal autoimmunity. Gastroenterology. 2008;134(4):1028–37. 10.1053/j.gastro.2008.01.070 18395084

[51] Yip L, Su L, Sheng D, Chang P, Atkinson M, Czesak M, et al Deaf1 isoforms control the expression of genes encoding peripheral tissue antigens in the pancreatic lymph nodes during type 1 diabetes. Nat Immunol. 2009;10(9):1026–33. 10.1038/ni.1773 19668219 PMC2752139

[52] Cohen JN, Guidi CJ, Tewalt EF, Qiao H, Rouhani SJ, Ruddell A, et al Lymph node-resident lymphatic endothelial cells mediate peripheral tolerance via Aire-independent direct antigen presentation. J Exp Med. 2010;207(4):681–8. 10.1084/jem.20092465 20308365 PMC2856027

[53] Nichols LA, Chen Y, Colella TA, Bennett CL, Clausen BE, Engelhard VH. Deletional self-tolerance to a melanocyte/melanoma antigen derived from tyrosinase is mediated by a radio-resistant cell in peripheral and mesenteric lymph nodes. J Immunol. 2007;179(2):993–1003. 17617591 10.4049/jimmunol.179.2.993

[54] Lee JW, Epardaud M, Sun J, Becker JE, Cheng AC, Yonekura AR, et al Peripheral antigen display by lymph node stroma promotes T cell tolerance to intestinal self. Nat Immunol. 2007;8(2):181–90. 10.1038/ni1427 17195844

[55] Carrega P, Loiacono F, Di Carlo E, Scaramuccia A, Mora M, Conte R, et al NCR(+)ILC3 concentrate in human lung cancer and associate with intratumoral lymphoid structures. Nat Commun. 2015;6:8280 10.1038/ncomms9280 26395069

[56] Mebius RE. Organogenesis of lymphoid tissues. Nat Rev Immunol. 2003;3(4):292–303. 10.1038/nri1054 12669020

[57] Scandella E, Bolinger B, Lattmann E, Miller S, Favre S, Littman DR, et al Restoration of lymphoid organ integrity through the interaction of lymphoid tissue-inducer cells with stroma of the T cell zone. Nat Immunol. 2008;9(6):667–75. 10.1038/ni.1605 18425132

[58] Barone F, Nayar S, Campos J, Cloake T, Withers DR, Toellner KM, et al IL-22 regulates lymphoid chemokine production and assembly of tertiary lymphoid organs. Proc Natl Acad Sci U S A. 2015;112(35):11024–9. 10.1073/pnas.1503315112 26286991 PMC4568258

[59] Corneth OB, Reijmers RM, Mus AM, Asmawidjaja PS, van Hamburg JP, Papazian N, et al Loss of IL-22 inhibits autoantibody formation in collagen-induced arthritis in mice. Eur J Immunol. 2016. 10.1002/eji.201546241 27067635

[60] Lenz G, Wright G, Dave SS, Xiao W, Powell J, Zhao H, et al Stromal gene signatures in large-B-cell lymphomas. N Engl J Med. 2008;359(22):2313–23. 10.1056/NEJMoa0802885 19038878 PMC9103713

[61] Fujisaka M, Ohtani O, Watanabe Y. Distribution of lymphatics in human palatine tonsils: a study by enzyme-histochemistry and scanning electron microscopy of lymphatic corrosion casts. Arch Histol Cytol. 1996;59(3):273–80. 8874759 10.1679/aohc.59.273

