# Supplementary figures and images for: A Reproducible Method for Isolation and In Vitro Culture of Functional Human Lymphoid Stromal Cells from Tonsils
Source: PLoS One. 2016 Dec 1;11(12):e0167555. doi: 10.1371/journal.pone.0167555 (PMC5132289; doi:10.1371/journal.pone.0167555)

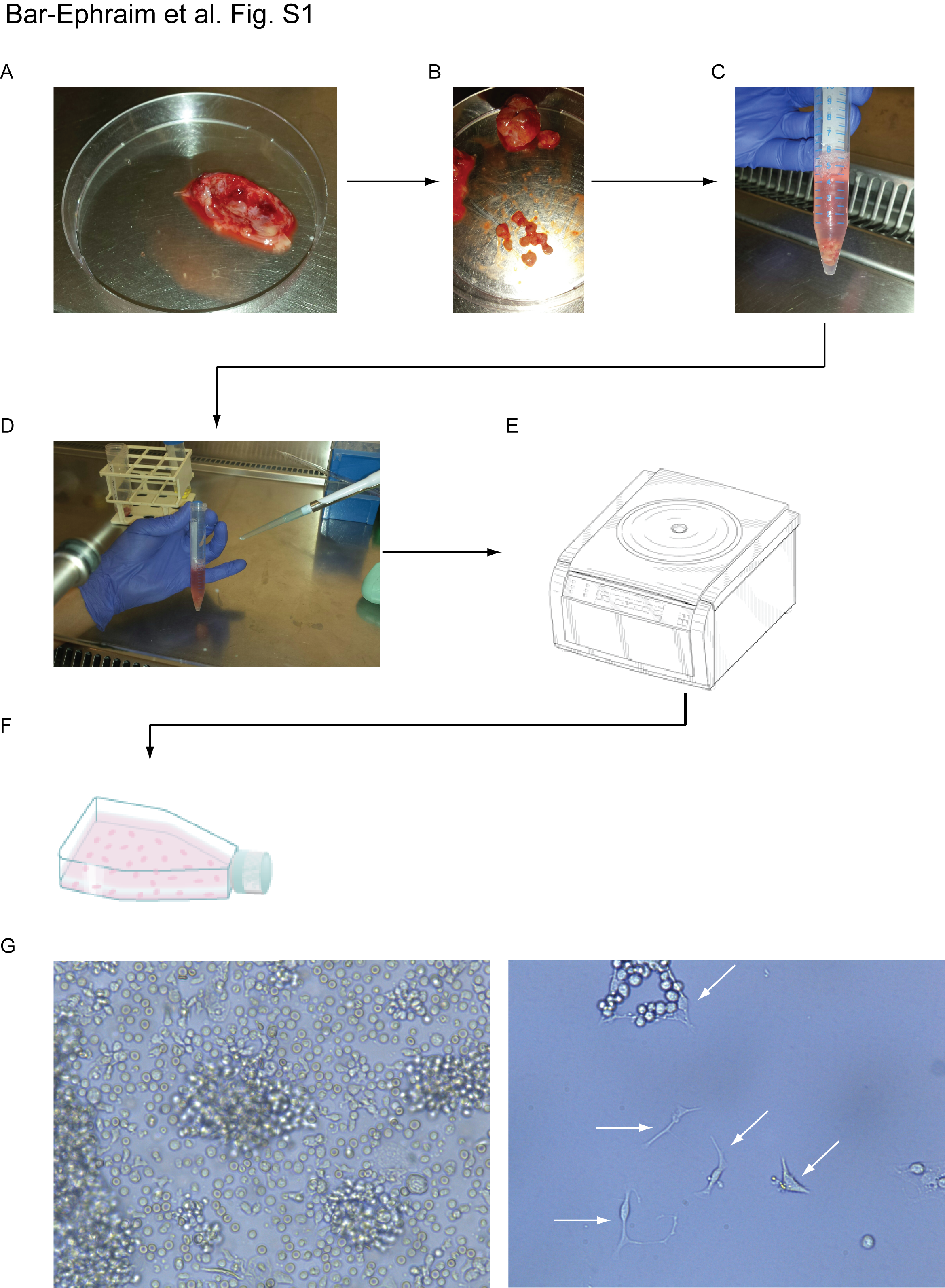

Supplement: S1 Fig — (A) 8–10 pieces of about 0.5X0.5 cm were cut from different parts of tonsils (B). The pieces of tonsil were incubated in RPMI supplemented with 0.6mg/mL collagenase P, 2.4mg/mL dispase and 0.3mg/mL DNAse I for 5 min. (C) after which they were disrupted using a 1000μL pipette (D). Subsequently, the supernatant was transferred to a tube containing PBS supplemented with 1% hi FCS and 2mM EDTA and centrifuged (E). Fresh enzyme containing medium was added to the pieces of tonsils and the process was repeated 4–5 times. Finally, the cells were centrifuged, taken up in culture medium and cultured in a collagen-coated T75 culture flask at 37°C / 5%CO2 (F). (G) Images of cultured tonsil cell suspensions 24hr. upon isolation before (left) and after (right) extensive washing with PBS. Images are representative of 13 independent isolations. (TIF) [file pone.0167555.s001.tif]

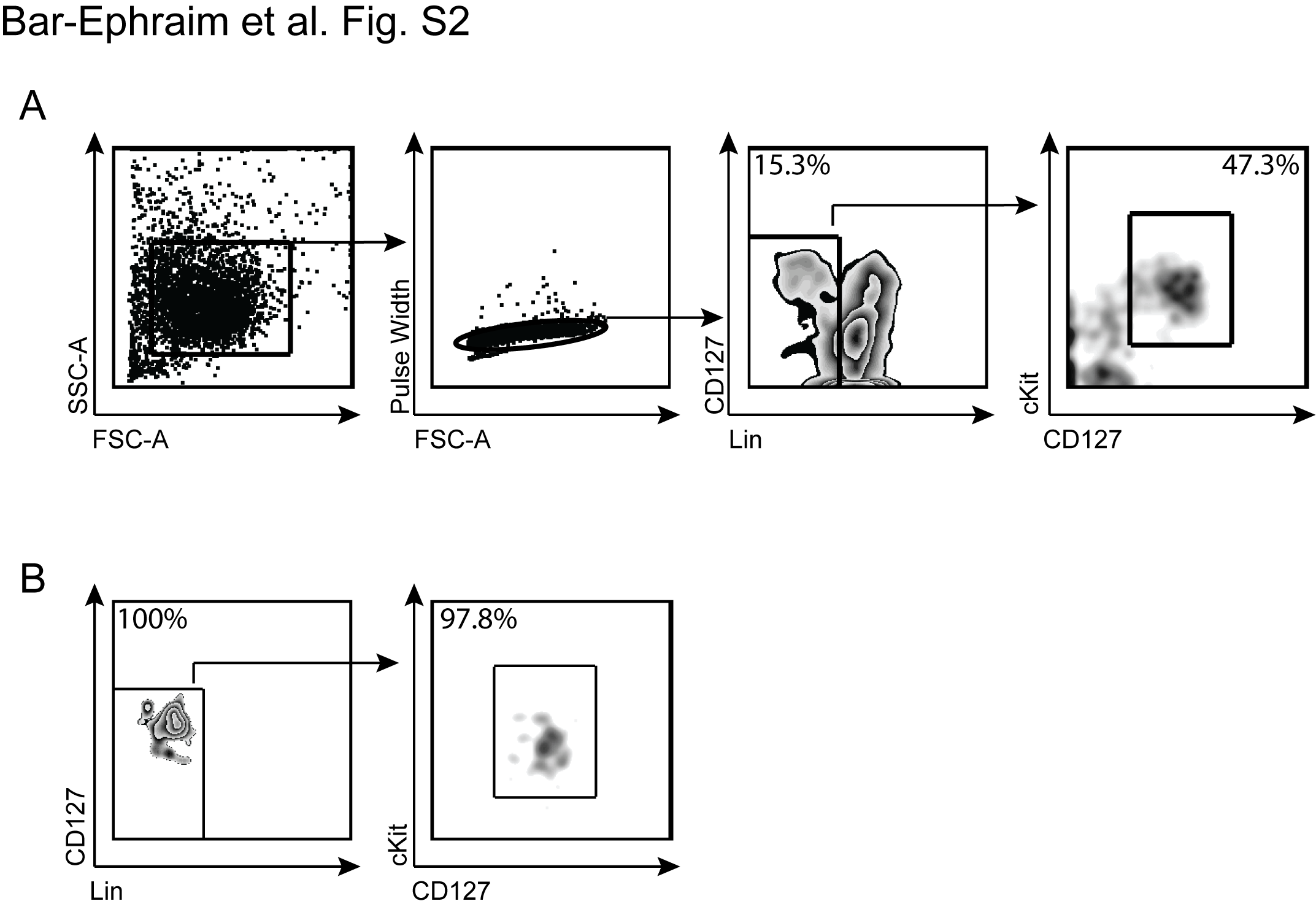

Supplement: S2 Fig — (A) ILCs were sort purified from cKit+ enriched fractions of human tonsils or peripheral blood as follows: lymphocytes were gated based on FSC-A vs SSC-A profile, after which doublets were excluded. Lin- cells were subsequently gated and finaly ILC3 were sorted as CD127+cKit+ cells. The lineage cocktail included monoclonal antibodies for human CD3, CD11c, CD14, CD19, CD34 and CD94. (B) Reanalysis of sorted cells. Images are representative of 5 independent sorts. In all plots: numbers represent percentage of cells within gate as part of all cells in the plot. (TIF) [file pone.0167555.s002.tif]
